# Detection of Methane Eructation Peaks in Dairy Cows at a Robotic Milking Station Using Signal Processing

**DOI:** 10.3390/ani12010026

**Published:** 2021-12-23

**Authors:** Ali Hardan, Philip C. Garnsworthy, Matt J. Bell

**Affiliations:** 1 School of Biosciences, Sutton Bonington Campus, The University of Nottingham, Loughborough LE12 5RD, UK; phil.garnsworthy@nottingham.ac.uk; 2 Agriculture Department, Hartpury University, Gloucester GL19 3BE, UK; matt.bell@hartpury.ac.uk

**Keywords:** cattle, methane, measurements

## Abstract

**Simple Summary:**

The objective of this study was to investigate the use of signal processing to detect eructation peaks in methane (CH_4_) released by dairy cows during robotic milking using three gas analysers. This study showed that signal processing can be used to detect CH_4_ eructations and extract spot measurements from individual cows whilst being milked. There was a reasonable correlation between the gas analysers studied. Measurement of eructations using a signal processing approach can provide a repeatable and accurate measurement of enteric CH_4_ emissions from cows with different gas analysers.

**Abstract:**

The aim of this study was to investigate the use of signal processing to detect eructation peaks in CH_4_ released by cows during robotic milking, and to compare recordings from three gas analysers (Guardian SP and NG, and IRMAX) differing in volume of air sampled and response time. To allow comparison of gas analysers using the signal processing approach, CH_4_ in air (parts per million) was measured by each analyser at the same time and continuously every second from the feed bin of a robotic milking station. Peak analysis software was used to extract maximum CH_4_ amplitude (ppm) from the concentration signal during each milking. A total of 5512 CH_4_ spot measurements were recorded from 65 cows during three consecutive sampling periods. Data were analysed with a linear mixed model including analyser × period, parity, and days in milk as fixed effects, and cow ID as a random effect. In period one, air sampling volume and recorded CH_4_ concentration were the same for all analysers. In periods two and three, air sampling volume was increased for IRMAX, resulting in higher CH_4_ concentrations recorded by IRMAX and lower concentrations recorded by Guardian SP (*p* < 0.001), particularly in period three, but no change in average concentrations measured by Guardian NG across periods. Measurements by Guardian SP and IRMAX had the highest correlation; Guardian SP and NG produced similar repeatability and detected more variation among cows compared with IRMAX. The findings show that signal processing can provide a reliable and accurate means to detect CH_4_ eructations from animals when using different gas analysers.

## 1. Introduction

Cattle are a notable source of CH_4_ emissions as a byproduct of rumen fermentation of food consumed. The animal releases CH_4_ generated in its rumen by eructation. A reliable direct measure of enteric CH_4_ from individual cows on commercial farms would allow more targeted emission mitigation on commercial farms, and the opportunity for farm level benchmarking and selection of low CH_4_ producing cows. A sniffer or breath sampling approach to measure enteric CH_4_ emissions from individual cows has shown great promise [1,2,3], due to the availability of portable gas analysis equipment and the finding that frequent CH_4_ measurement during robotic milking has a high correlation (r = 0.89) with respiration chamber measurements of total CH_4_ production from the same cows [1]. Frequent “spot’” measurements of CH_4_ taken within a day and expressed as area under CH_4_ peaks, mean concentration, or ratio of CH_4_ to CO_2_, produce repeatable measurements [3,4,5]. For reliable measurements, however, potential sources of error, such as head position of the cow [4] and number of measurements obtained [6,7], need to be taken into account. The position of a cow’s head relative to the gas sampling point can be indicated by a proximity sensor [4]. An alternative solution is to employ advanced data filtering methods to identify eructation peaks of CH_4_ [1]. Methods that are portable, non-invasive, and do not change the cow’s normal routine or surroundings, such as the technique used in this study, are of great interest.

Gas analysers produce an electric signal that is then converted to a measure of gas concentration. Peaks in the signal of CH_4_ concentration represent eructations when sampling emissions from the mouth and nostrils of cows. The eructation peaks differ in terms of frequency, height, and rise time of the peak. The current study builds on previous research [8], which recommended using the maximum amplitude of an eructation to quantify CH_4_ emissions. By extracting the amplitude of eructation peaks, the background CH_4_ in the environment is removed. The hypothesis of the current study was that enhanced filtering of eructation spot measurements using signal processing could be used with different gas analysers and would provide a repeatable and reliable measure for commercial farm use. The objectives of the current study were to investigate the use of signal processing to detect eructation peaks of CH_4_ released by individual cows during robotic milking, and to compare recordings from three gas analysers differing in volume of air sampled and response time.

## 2. Materials and Methods

Approval for this study was obtained from the University of Nottingham Animal Welfare and Ethical Review Board before commencement of the study (approval number P78FDB0C3).

### 2.1. Data

Concentrations of CH_4_ in breath was measured during milking of 65 Holstein-Friesian dairy cows at the Nottingham University Centre for Dairy Science Innovation (Sutton Bonington, Leicestershire, UK). The dataset included cows with a wide range of values for lactation number and stage of lactation, milkings per day, milk yield, and live weight (Table 1).

Cows were housed in one pen of a freestall barn and individually milked in a robotic milking station (Lely Astronaut A4; Lely UK Ltd., St Neots, UK). Gas concentrations (*v*/*v*) were measured every second in air sampled continuously from the feed bin of the robotic milking station using three infrared gas analysers sampling air concurrently: Guardian SP and Guardian NG (Edinburgh Instruments Ltd., Livingston, UK; both T90 response time < 30 s) at 0.75 L/min and IRMAX (Crowcon Ltd., Abingdon, UK; T90 response time < 4 s) at 0.75 L/min in period 1, 375 L/min in period 2, and 750 L/min in period 3 (representing an increase in air speed from 0.5 m/s in period 1 to 5 m/s in period 3). The IRMAX gas analyser allowed adjustment of its air sampling volume as stated, whereas the volume of sampled air by both the Guardian SP and NG analysers cannot be adjusted.

**Figure 1:**
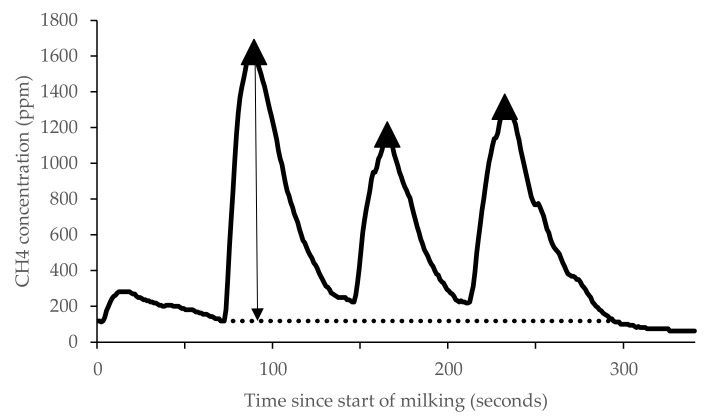
The CH4 concentration profile in eructated gas for a single cow during milking showing measured peaks (▲) and measurements for maximum peak amplitude (solid black line with arrow).

The CH_4_ concentration (*v*/*v*) measured by each analyser was recorded at 1 s intervals on a data logger (Simex SRD-99; Simex Sp. z o.o., Gdańsk, Poland) and visualised using logging software (Loggy Soft; Simex Sp. z o.o, Gdańsk, Poland). The CH_4_ concentration during milking was recorded in parts per million (*v*/*v*) (Figure 1). Then CH_4_ concentration data were extracted from the time-series signal using the peak analysis tools in MATLAB Signal Processing Toolbox (version R2018a, The MathWorks, Inc., Natick, MA, USA. See [9] for metrics). Peak analysis tools were used to identify eructation peaks and extract the maximum amplitude within each milking from raw logger data for the analysis.

Measurements of enteric CH_4_ during milking were conducted during 3 consecutive sampling periods of 7 days, in which cows were fed the same commercial partial mixed ration of 50% forage (grass silage, maize silage, and wholecrop wheat) ad libitum plus 50% concentrates on a dry matter basis. The chemical composition of the partial mixed ration was dry matter, 46.3%; Metabolisable Energy (ME), 12.0 MJ; Crude Protein (CP), 17.5%; Neutral-detergent Fibre (NDF), 36.7%; starch, 16.3%; sugars, 6.7%; and fat, 3.7% (analysed by a commercial analytical laboratory (Sciantec analytical, Cawood, UK)). Additional concentrates were fed during milking at a daily allowance of 1.5 kg plus 0.16 kg per litre of milk yield above 23 L/d. Concentrate was dispensed into the feed bin throughout the milking period, which helps keep the cow’s head within suitable proximity of the gas sampling tube. Concentrate manufacturer’s declared specification per kilogram as fed was dry matter, 88%; ME, 12.2 MJ; CP, 16%; NDF, 24%; starch, 21%; and fat, 6.2%. Milk yield, live weight, and concentrate intake were recorded automatically at each milking. The total dry matter intake of the cows was not measured. A total of 5512 CH_4_ concentration measurements (averaging 134 ± 54 records per cow from the three analysers) were obtained during milkings from 65 cows during 3 consectutive sampling periods.

### 2.2. Statistical Analysis

Methane measurements were analysed using a linear mixed model in Genstat Version 19.1 (Lawes Agricultural Trust, Harpenden, UK, 2018). Average CH_4_ concentration per week per cow was used for analysis and provided 504 individual cow CH_4_ records (3 analysers × 3 weeks × 56 cows). Equation (1) was used to calculate predicted mean values and variance components for CH_4_ per cow:

y_ijkl_ = µ + P_i_ × A_j_ + L_k_ + *β*DIM + C_l_ + E_ijkl_ (1)

where y_ijkl_ is the dependent variable; µ is the overall mean; P_i_ is the fixed effect of sampling period (periods 1, 2 or 3); A_j_ is the fixed effect of analyser (SP, NG or IRMAX); L_k_ is the fixed effect of lactation number (1, 2, or 3 and more); *β*DIM is the linear regression of Y on days in milk; C_l_ is random effect of individual cow; E_ijkl_ is the residual error term.

Pearson correlation coefficient (r) was used to assess the association between CH_4_ measurements from SP, NG, and IRMAX gas analysers. Repeatability of gas concentration measures were assessed by σ^2^ animal/(σ^2^ animal + σ^2^ residual), where σ^2^ is the variance. Between-cow and residual coefficients of variation (CV) were calculated from variance components as root mean square error divided by the mean. Significance was attributed at *p* < 0.05.

## 3. Results

**Figure 2:**
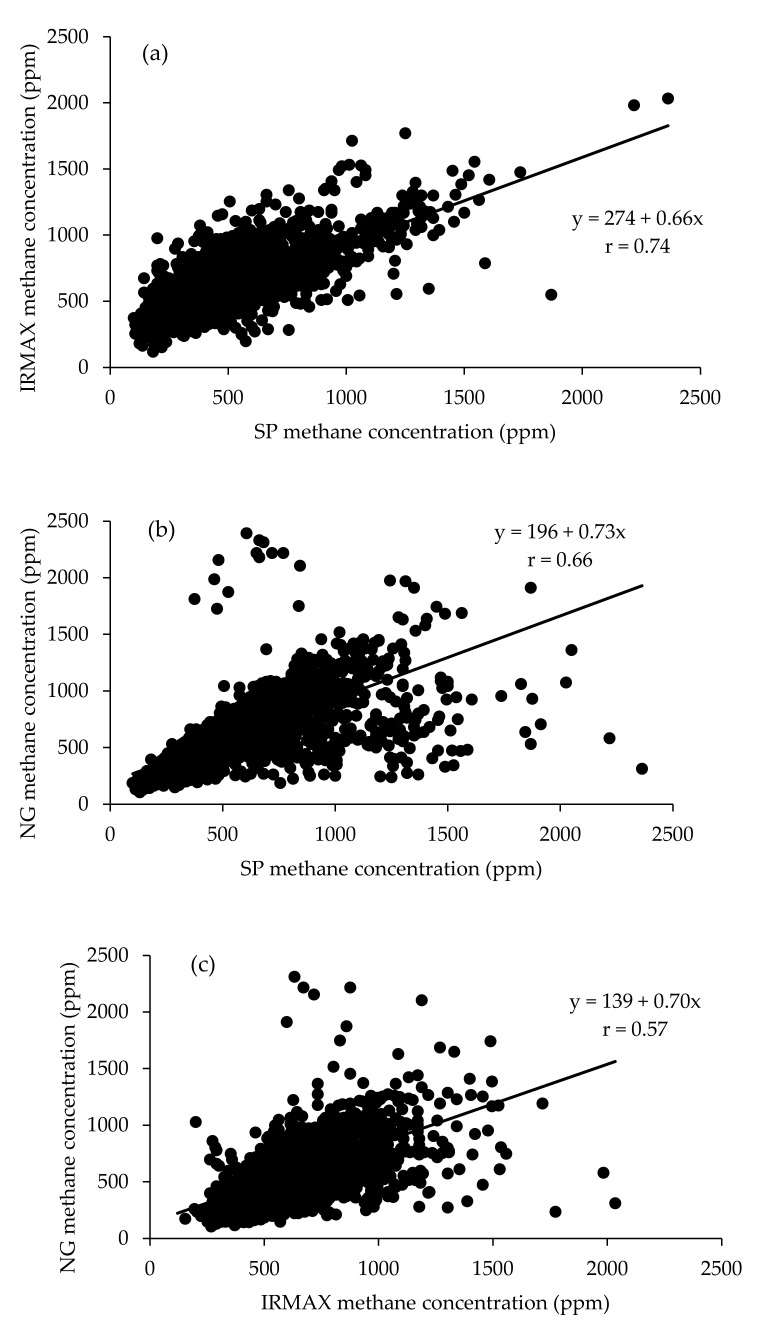
The relationship between maximum eructation peaks measured by (**a**) IRMAX and SP gas analysers, (**b**) Guardian NG and SP gas analysers, and (**c**) IRMAX and NG gas analysers during the study period.

In the current study, three analysers were compared. There was a positive correlation between CH_4_ measurements from individual milkings obtained using the three gas analysers (r = 0.57 to 0.74) (Figure 2a–c). Measurements from the SP and IRMAX analysers had the highest correlation (r = 0.74; Figure 2a).

At the same air sampling volume (0.75 L/min) in period one, there was no difference in measured CH_4_ concentration between the analysers (Table 2). Increasing the volume of air sampled by the IRMAX in periods two and three resulted in a higher concentration measured by the IRMAX and a lower concentration measured by the SP (*p* < 0.001), particularly in period three, but no change in the average concentration measured by the NG across periods.

The repeatability of CH_4_ measurements by SP (0.59) and NG (0.60) were similar and higher than IRMAX (0.52) (Table 3). Also, the between-cow CV were higher for SP (0.20) and NG (0.18) than for IRMAX (0.16). Residual CV were similar across analysers during all three periods in the range 0.06–0.10 (Table 3).

## 4. Discussion

The use of mobile gas analysers to measure CH_4_ emissions from large numbers of animals across populations is of great interest given its adaptability to normal farm environments and non-invasive setup [10]. There are several challenges to obtaining a repeatable and precise measure of enteric CH_4_ in the farm environment using non-invasive methods, such as the frequency of measurements [6], head position of the animal relative to the sampling tube [4] and obtaining a concentration measure from often noisy data [8,10,11]. On average, the cows in the current study provided 2.1 spot measurements per day using each gas analyser and were measured for seven days, which is required to provide a sufficient number of measurements for comparisons [1]. The current study applied a novel approach of signal processing to detect eructation peaks. Signal processing is widely used in the fields of medical care or audio detection based on electrical or noise waves. However, for animal monitoring, this application is new but appropriate for potentially noisy data when measuring gas concentrations in animal breath. The proximity of the animals’ head to the sampling tube was not measured but the approach used in the current study assumes that the eructation produced by a cow, and with the greatest amplitude during a milking, represents the time when the mouth and nostrils of the cow are closest to the sampling tube. This approach therefore accounts for cow head position to obtain a representative spot measurement from the animal being sampled. This approach to eructation peak detection is based on the theory that CH_4_ pulses expelled by the animal can produce a repeatable and reliable measure of individual CH_4_ emissions from spot measurements when compared to respiration chamber measurements [1,8,12].

Measuring enteric CH_4_ emissions using eructation peaks in concentration has been shown to provide a highly repeatable phenotype for ranking cows on CH_4_ emissions [10]. In this study, the repeatability of average CH_4_ measurement from eructation amplitude ranged from 0.52 for IRMAX to 0.60 for NG, which are similar to values reported in previous studies ranging from 0.44 to 0.87 using other breath measurement techniques (e.g., Sulphur hexafluoride tracer, Greenfeed, portable infrared analysers for gas flux or concentration; [3,4,8,10]). The similar repeatability of the SP and NG analysers may be explained by their common sampling volume of 0.75 L/min and manufacturer. These mobile gas analysers taking spot measurements have been found to be more repeatable than chamber CH_4_ measurements [8,10] and particularly short-term spot measurements (e.g., Laser methane detector; [10]), presumably due to the range in the stage of production of the animals and possibly their behaviour when using such sampling approaches. Even with the similarities between the SP and NG performance, the SP and IRMAX measurements were more correlated. The NG measurements were more variable, which might be explained by the ability of the machine to work in a farm environment with changes in air moisture and temperature. Only a single dust filter was used in the sampling line of each analyser. Between-cow CV values found in the current study (ranging from 0.16 to 0.20) were similar to values reported in other studies using the sniffer approach (ranging from 0.10 to 0.21) but are often observed to be higher than values reported for daily CH_4_ emissions in studies using respiration chambers (range of 0.08 to 0.09) [8,10,13]. Residual CV values found in the current study were similar (ranging from 0.06 to 0.10) to values reported in other studies using the sniffer approach (ranging from 0.01 to 0.09 respectively) and for daily CH_4_ emissions in chamber studies (range of 0.03 to 0.12) [8,10,13].

The current study supports the need for a reliable method to enhance signal filtering [14]. Signal processing can be used with different gas analyzers at different airflow rates and contribute to enhanced eructation detection.

## 5. Conclusions

This study compared three infrared gas analysers for measuring enteric CH_4_ from dairy cows during milking on a farm, using signal processing to detect eructations. When sampling the same volume of air, all gas analysers measured similar CH_4_ concentrations. Across the periods studied, measurements by the SP and IRMAX analysers had the highest correlation, but the SP and NG analysers had similar repeatability, and the between-cow and residual CV values compared to the IRMAX analyser. The findings show that signal processing can provide a reliable and accurate means to detect CH_4_ eructations from animals when using different gas analysers.

## Figures and Tables

**Table 1:** Average production values for cows in the study (*n* = 65).

Item	Units	Mean (s.d.) ^1^	Range
Lactation	no.	1.9 (1.3)	1–7
Stage of lactation	days	154 (83)	11–350
Milkings	per day	2.8 (0.9)	1–5
Milk yield	kg/day	42 (11)	5–80
Live weight	kg	722 (83)	499–911

^1^ s.d. = standard deviation.

**Table 2:** Effect of analyser (SP, NG or IRMAX) and period on methane concentration (ppm). Means with different superscript letters in the same row differ (*p* < 0.05).

Variable	Mean									F Statistic	SED	*p*-Value
Analyser	SP	NG	IRMAX									
	545 ^a^	595 ^b^	621 ^c^							37.2	9.1	<0.001
Period	1	2	3									
	582	594	585							1.2	9.9	0.303
Analyser × Period ^1^	SP P1	SP P2	SP P3	NG P1	NG P2	NG P3	IRMAX P1	IRMAX P2	IRMAX P3			
	574 ^ab^	544 ^bc^	516 ^c^	600 ^a^	596 ^a^	590 ^a^	571 ^a^	643 ^d^	650 ^d^	10.6	16.1	<0.001

^1^ SP = Guardian SP; NG = Guardian NG; IRMAX = IRMAX analyser; P1 − P3 = periods 1 to 3.

**Table 3:** Repeatability of methane measurements and coefficients of variation (CV) for three gas analysers.

		Analyser	
Statistic	SP	NG	IRMAX
Repeatability	0.59	0.60	0.52
Between-cow CV	0.20	0.18	0.16
Residual CV			
Period 1	0.10	0.09	0.08
Period 2	0.06	0.06	0.07
Period 3	0.09	0.08	0.08

## Data Availability

The datasets analysed are available from the corresponding author on request.

## References

[1] Garnsworthy P.C., Craigon J., Hernandez-Medrano J., Saunders N. (2012). On-farm methane measurements during milking correlate with total methane production by individual dairy cows. J. Dairy Sci..

[2] Lassen J., Løvendahl P. (2016). Heritability estimates for enteric methane emissions from Holstein cattle measured using noninvasive methods. J. Dairy Sci..

[3] Bell M.J., Potterton S., Craigon J., Saunders N., Wilcox R., Hunter M., Goodman J., Garnsworthy P.C. (2014). Variation in enteric methane emissions among cows on commercial dairy farms. Animal.

[4] Huhtanen P., Cabezas-Garcia E., Utsumi S., Zimmerman S. (2015). Comparison of methods to determine methane emissions from dairy cows in farm conditions. J. Dairy Sci..

[5] Negussie E., Lehtinen J., Mäntysaari P., Bayat A.-R., Liinamo A.-E., Mäntysaari E.A., Lidauer M. (2017). Non-invasive individual methane measurement in dairy cows. Animal.

[6] Cottle D., Velazco J., Hegarty R., Mayer D. (2015). Estimating daily methane production in individual cattle with irregular feed intake patterns from short-term methane emission measurements. Animal.

[7] Hammond K.J., Crompton L.A., Bannink A., Dijkstra J., Yáñez-Ruiz D.R., O’Kiely P., Kebreab E., Eugène M., Yu Z., Shingfield K.J. (2016). Review of current in vivo measurement techniques for quantifying enteric methane emission from ruminants. Anim. Feed. Sci. Technol..

[8] Bell M.J., Garnsworthy P.C., Mallis D. (2020). Modified approach to estimating daily methane emissions of dairy cows by measuring filtered eructations during milking. J. Sustain. Org. Ag. Syst..

[9] MathWorks Peak Analysis. https://uk.mathworks.com/help/signal/examples/peak-analysis.html.

[10] Garnsworthy P.C., Difford G.F., Bell M.J., Bayat A.R., Huhtanen P., Kuhla B., Lassen J., Peiren N., Pszczola M., Sorg D. (2019). Comparison of Methods to Measure Methane for Use in Genetic Evaluation of Dairy Cattle. Animal.

[11] Bell M.J., Saunders N., Wilcox R., Homer E., Goodman J., Craigon J., Garnsworthy P.C. (2014). Methane emissions among individual dairy cows during milking quantified by eructation peaks or ratio with carbon dioxide. J. Dairy Sci..

[12] Crompton L., Mills J., Reynolds C., France J., Sauvant D., van Milgen J., Faverdin P., Friggens N. (2011). Fluctuations in methane emission in response to feeding pattern in lactating dairy cows. Modelling Nutrient Digestion and Utilisation in Farm Animals.

[13] Huhtanen P., Krizsan S., Hetta M., Gidlund H., Cabezas Garcia E. Repeatability and between cow variability of enteric methane and total carbon dioxide emissions. Proceedings of the Greenhouse Gases in Animal Agriculture conference, Advances in Animal Biosciences.

[14] Sorg D., Difford G.F., Mühlbach S., Kuhla B., Swalve H.H., Lassen J., Strabel T., Pszczola M. (2018). Comparison of a laser methane detector with the GreenFeed and two breath analysers for on-farm measurements of methane emissions from dairy cows. Comput. Electron. Agric..

